# Molecular Effects of Physical Activity and Body Composition: A Systematic Review and Meta-Analysis

**DOI:** 10.3390/ijerph22040637

**Published:** 2025-04-18

**Authors:** Jenni Chambers, Clare M. P. Roscoe, Corinna Chidley, Agnieszka Lovett, Aparna Duggirala

**Affiliations:** 1 Biomedical and Clinical Sciences, School of Science, University of Derby, Derby DE22 1GB, UK; j.chambers1@derby.ac.uk (J.C.); 2 Clinical Exercise Rehabilitation Research Centre, School of Sport and Exercise Science, University of Derby, Derby DE22 1GB, UK; c.chidley@derby.ac.uk

**Keywords:** epigenetics, genetics, biomarker, methylation, CpG, obesity, physical activity, body composition, BMI, body mass index

## Abstract

Physical activity (PA) and body composition are important lifestyle factors that influence public health. Research suggests that DNA regions (CpG site locations) are differentially methylated in a physically active population. This meta-analysis aimed to identify CpG sites associated with various levels of PA and associated metabolic pathways. The meta-analysis followed PRISMA guidelines using PubMed, SportDISCUS, Embase, Scopus, Cochrane and Web of Science. Epigenomic analyses performed on DNA of participants with no underlying health conditions were included. Articles were screened using Rayyan AI and extracted CpG sites, and their location were confirmed using the EWAS catalogue. Six studies comprising 770 subjects were included in this meta-analysis. The meta-analysis was performed on clinical metrics extracted from the six studies and showed that BMI, blood pressure, insulin and glucose testing are significantly improved upon PA intervention. Amongst the included studies, a total of 257 CpG sites were differentially methylated in physically active participants, with 134 CpGs located in 92 genes associated with obesity-related pathways. The identified differentially methylated genes either belonged to the lipid metabolism or insulin signalling pathway. The genes which were differentially regulated in multiple tissue types and studies are JAZF1 (insulin signalling, and lipid and carbohydrate metabolism pathways) and NAV1 (mTOR signalling pathway). In conclusion, the current epigenomic meta-analysis showed that PA levels induce differential DNA methylation signatures on genes that affect metabolism. To understand the positive molecular effects of PA, further research on the above candidate genes needs to be conducted amongst various levels of a physically active population.

## 1. Introduction

Obesity is a significant global health problem. It affects one in eight adults, and this figure has doubled since 1990; 2.5 billion adults are overweight and 890 million are living with obesity worldwide [1]. Obesity places a significant burden on healthcare resources and society, as it plays a contributing factor to numerous preventable diseases, including type II diabetes (T2D), cardiovascular disease (CVD) and cancer.

Weight range classifications published by the National Institute for Health and Care Excellence (NICE) are used to identify risk categories based on body mass index (BMI) measurements, using the formula weight (kg)/height (m)^2^. The threshold for those individuals described as overweight is BMI ≥ 25 kgm^2^, and for those living with obesity is BMI ≥ 30 kgm^2^ [2]. BMI is a commonly used measure as it is quick and simple to calculate, requiring only basic equipment for height and weight measurement; however, it has some limitations [3]. Additional health indicators are increasingly being used alongside BMI, such as waist circumference (WC), waist to hip ratio (WHR), body fat percentage and lipid profiles, to provide a broader indication of health and body composition [4]. Recent recommendations suggest that BMI alone should not be used to confirm excess adiposity or as an individual measure of health, but should be used alongside other parameters that better represent distribution of fat, such as WHR [5].

Physical activity (PA) is recommended by NICE [2] as a weight management approach. PA is a broad term covering any bodily movement produced by skeletal muscles requiring energy expenditure; this could be during leisure time, travel, or as part of a person’s work or domestic activities [6]. Exercise is a form of PA, and goes a step further, describing planned, structured, repetitive bodily movement, intended to improve or maintain components of physical fitness [7]. The UK Chief Medical Officer has published recommendations on weekly PA levels for a healthy lifestyle. For an adult aged 18+ years, this includes a minimum of 150 min of moderate or 75 min of vigorous PA per week, including strength building activities on at least two days per week and minimising sedentary time [8]. The International Physical Activity Questionnaire (IPAQ) [9] describes vigorous activity as requiring hard physical effort, resulting in harder breathing than normal, with examples including aerobics, heavy lifting or fast cycling; moderate activity is described as requiring moderate physical effort with somewhat harder breathing than normal, and examples include carrying light loads or cycling at a regular pace; walking could be considered light exercise. Sedentary behaviour (SB) is described as waking time spent sitting or lying, with low energy expenditure. SB contributes significantly to obesity related diseases including T2D and CVD, and metabolic syndrome [10]; it is positively associated with adipose tissue insulin resistance, even where there is also moderate-to-vigorous PA taking place, particularly in those with higher BMI [11]. A distinction should be made between a sedentary person and SB, as an individual who is physically active may also display SB, and an individual with low levels of PA may not display SB [12].

PA is known to have beneficial effects on overall health and weight loss, quality of life and disease prevention [13]. Variations in individual responses to PA are influenced by multiple intrinsic factors including sex, age, race, ethnicity, genetics and epigenetics [14]. Genome-wide association studies (GWAS) have identified genetic variants linked with obesity, such as the FTO, MC4R, TMEM18, SEC16B and TFAP2B genes [15,16]. Epigenetic changes are influenced by lifestyle factors, such as diet and level of PA. These changes include DNA methylation (DNAm), micro-RNA expression and histone and chromatin modifications. They affect transcription processes, gene expression and function without affecting the DNA sequence itself. Improved understanding of molecular changes resulting from PA at a DNAm level indicates further health results, such as chronic disease risk reduction [17], which are not observed using clinical measures. The complex relationship between PA and body composition (BC) needs further exploration, as PA acts as an external epigenetic factor influencing DNAm [18], presenting an interesting question of epigenetic cause and effect in weight loss and obesity [19].

DNAm changes are affected by DNA methyltransferase enzymes acting in response to PA, including methylenetetrahydrofolate reductase, methionine synthase and methionine synthase reductase, which provide methyl groups to DNA as part of the metabolic cycle [20]. As DNAm is a regulator of gene expression, an increase in DNAm (hypermethylation) in cytosine–phosphate–guanine groups (CpG site) within a promotor region is associated with a decrease in transcription [21], which can inhibit gene expression and ultimately silence or switch off the gene. Conversely, a decrease in DNAm of CpG sites (hypomethylation) may enable increased gene expression [22] or lead to genetic instability through reduced gene regulation. Effects may vary depending on the function of the gene affected and any pathways it is associated with. For example, if PA induces DNAm changes in genes and pathways associated with metabolism or disease, this may indicate a beneficial or protective effect of PA [21].

Observational studies have identified variations in the DNAm profiles of those with habitual PA routines versus those considered inactive, affecting epigenetic age compared to biological age as an indicator of health and mortality markers [23]; a higher epigenetic age in sedentary populations can be reduced following the introduction of PA [24]. Differences in DNAm profiles have also been observed in individuals classed as obese versus those considered a healthy weight [25] and following PA interventions [17]; targeted gene analysis found altered DNAm levels in genes associated with obesity following PA [26,27]. Epigenome-wide association studies (EWAS) in monozygotic twins found a correlation between BMI and methylation of genes associated with obesity [28], and that environmental and PA factors influenced epigenetic alterations associated with metabolic risk factors [29]. This indicates that PA can exert an epigenetic influence on genes associated with obesity and metabolism even where there is genetic homogeneity.

DNAm responses to PA vary according to tissue type [30]; blood is commonly used for DNAm analysis as it can be collected using less invasive methods than other tissue types. Skeletal muscle (SKM) and adipose tissue (AT) are often obtained through biopsy for use in epigenetic analysis relating to PA. Other factors causing variation in epigenetic responses to PA include sex, with differential physiological responses in males and females [31], and age, as epigenetic changes accumulate over the life span [32]. However, epigenetic profiles are both heritable and reversible [33] which suggests that lifestyle changes can influence DNAm profiles [34].

Several independent whole EWAS are available on understanding the molecular link between PA and metabolic health. It is imperative to perform a comprehensive epigenomic meta-analysis to find the molecular link and metabolic pathway replicated in all the EWAS published. Therefore, this systematic review aims to compile a comprehensive data set and meta-analysis detailing differentially methylated CpG sites resulting from PA and any associated genes and pathways related to obesity. The review will improve our understanding of how epigenetic profiles are affected by PA at specific CpG sites. This information may be used to inform more in-depth laboratory investigations into DNA methylation profiles resulting from PA, with an emphasis on understanding more about interactions between PA, epigenetics, obesity and related diseases.

## 2. Materials and Methods

### 2.1. Protocol and Registration

The details of this systematic review were registered with PROSPERO in November 2023. The review protocol can be accessed on the PROSPERO website using the registration number CRD42023471011 or through the following website address: https://www.crd.york.ac.uk/prospero/display_record.php?ID=CRD42023471011 (accessed on 28 January 2025).

This literature review was completed using the preferred reporting items for systematic reviews and meta-analyses (PRISMA) framework [35].

### 2.2. Study Selection Criteria

Eligibility criteria were established using the population, intervention, comparison, outcome and study (PICOS) framework (Table 1), to establish the PICOS design. All peer-reviewed articles in English, or that had been translated into English, up to the search date of 16 November 2023 were reviewed, and searches were updated on 5 September 2024.

Human-only study participants were recruited, between 18 and 65 years of age, male or female and otherwise healthy with no underlying health conditions. Studies using participants outside this age range were excluded. Excluded health conditions included diabetes type I, type II and those known to be pre-diabetic; autoimmune disorders such as multiple sclerosis; health complaints such as myalgic encephalomyelitis/chronic fatigue syndrome or long-COVID; clotting conditions such as haemophilia; blood-borne viruses such as human immunodeficiency virus or hepatitis; known genetic disorders; cardiovascular disease; cancer. Participants were also excluded if pregnant or breastfeeding, or on any prescribed medication. Studies focusing on elite athletes, military-trained personnel and smokers were excluded.

Studies that analysed DNAm of CpG sites based on PA levels were considered. This could have resulted from either an assessment of individual PA levels or a PA intervention. Controls for the PA intervention studies were either participant baseline measures taken pre-intervention or a comparable non-exercising group. Original epigenome-wide studies and targeted DNA methylation studies analysing multiple genes were considered. Studies including results for specific CpG sites relating to PA were selected; studies analysing only one gene were excluded; studies reporting results including confounding factors, e.g., diet, were excluded. Only studies using SKM, AT or blood samples were selected. Single case reports, expert opinion manuscripts, letters to the editor, commentaries, conference papers and review papers were excluded from the review.

### 2.3. Search Strategy

A range of online electronic databases were used for the literature search, including Pubmed (including Medline), SPORTDiscus, Embase, Scopus, Web of Science and Cochrane Library. All databases were last searched on 5 September 2024, using a combination of the following keywords within the titles: epigenetic, genetic, biomarker, methylation, DNAm, CpG, physical activity, physical exercise, PA, body composition, BMI, body mass index, waist circumference (Supplementary File Table S1).

Search results were exported from the databases and uploaded into Rayyan AI [36] to manage and track paper screening using titles and abstracts. Duplicates were automatically detected using system filters and manually checked and removed. Titles were screened for relevance against the search criteria. In cases where the scope of the study was unclear from the title, an additional abstract screen was undertaken, using the Rayyan AI system. Remaining articles which had not been excluded were then subject to a full-text screening.

**Figure 1:**
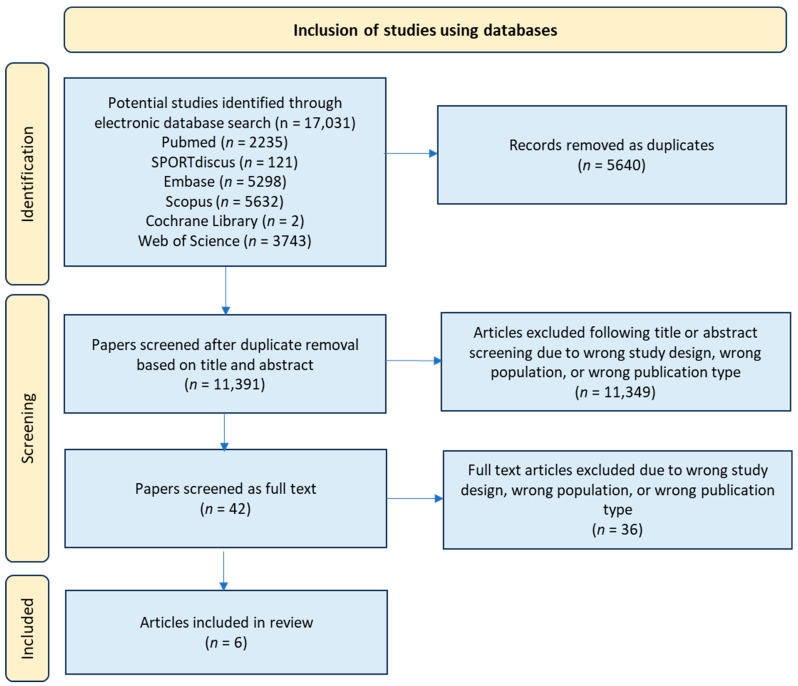
Preferred reporting items for systematic reviews and meta-analyses (PRISMA) framework flowchart, indicating inclusion and exclusion decisions. *n* = number of papers.

Details of the shortlisted papers were recorded in an Excel spreadsheet, including database accessed, DOI, publication, title, citation reference and comments on inclusion or exclusion decisions. This stage was completed by the lead researcher (J.C.). The shortlist was shared with a panel of second researchers (A.D., C.M.P.R., C.C.) to ensure agreement on articles selected for inclusion. The PRISMA search strategy can be viewed in Figure 1.

### 2.4. Study Quality Assessment

Papers selected for inclusion were quality-checked using the BIOCROSS method [37], a tool developed to evaluate biomarker studies. This was not intended as an additional selection step, but to critically evaluate the selected studies for risk of bias.

The BIOCROSS method comprised five domains of questions and sub-domains to examine study rationale, design and methods, data analysis, data interpretation and biomarker measurement. Each section contained three elements; if all three elements had been covered in the study, two points were awarded for that section; if one or two elements were not covered, one point was awarded. If no elements were covered, that section would achieve zero. A maximum score of 20 points was available, and studies with higher scores could be considered more robust and good quality with low risk of bias, while studies with lower scores would be considered less reliable with a higher risk of bias. No thresholds of acceptability were published at the time of evaluation (Supplementary File Table S2).

### 2.5. Analysis

Anthropomorphic characteristics and clinical metrics were compiled for each study using Excel version 2408. These factors included the size and characteristics of the cohorts and various measurements of health, fitness, body composition and classification of PA levels. Forest plots were created using Cochrane Review Manager (RevMan) web software version 9.0.0 [38] to determine the statistical significance of clinical metric changes post-PA intervention.

CpG sites associated with PA from studies meeting the eligibility and inclusion criteria were compiled into an Excel table, categorised by sample tissue type. Details included methylation percentage levels, M values or beta-values for individual CpG sites and *p*-values for significant differentials, where available. Associated genes or pathways identified in the studies were recorded. CpG sites were checked against the epigenome-wide association studies (EWAS) [39], Accessible Resource for Integrated Epigenomic Studies (ARIES) [40], Genetics of DNA Methylation Consortium (GoDMC) [41] and Kyoto Encyclopaedia of Genes and Genomes (KEGG) [42] databases for gene or pathway associations with obesity. Statistical analysis of the compiled CpG data was conducted using XY histogram plots in GraphPad Prism 10.4.1 for Windows (GraphPad Software, Boston, Massachusetts, USA, www.graphpad.com, accessed on 25 March 2025). Any agreements between data sets, such as consensus on CpGs, genes or pathways associated with obesity, were identified and summarised. Studies were cross-referenced to establish whether CpGs identified as significant in one study had been analysed and found not to be significant in another study. Finally, the discussion sections were reviewed to identify any common themes, findings or areas requiring further investigation, which could present opportunities for future research.

## 3. Results

### 3.1. Study Selection

A total of 17,031 articles were identified through the keyword search across the six electronic databases. Once uploaded into Rayyan, 5640 of these articles were automatically identified as duplicates by the software, so were screened by title by the researcher (J.C.) and selection decisions made with duplicates removed as one step. The remaining 11,391 articles were then screened based on title, with an additional abstract screening where more clarity was needed on the nature of the study. At this stage, 8568 papers were excluded due to not meeting the study design selection criteria, mostly because they were not DNA methylation studies or not specifically focused on PA. A total of 2538 studies were excluded for focusing on the wrong population, for example, using participants from the wrong age group, smokers, or with a specifically excluded condition or disease. A total of 243 articles were excluded due to being the wrong publication type, for example, conference proceedings or letters to the editor. Some articles fell into more than one of these exclusion categories. A total of 42 papers subsequently remained and were subject to a full-text screening (Figure 1).

During the full-text screening, 36 further articles were excluded, mostly for being the wrong study design or population. For example, some studies did not state the age group being studied in the abstract and fell outside of the age criteria upon full article screening [43]. Other studies mentioned PA in the abstract but did not specifically report on this in the results or separate it out from other factors such as diet [44,45,46]. Other articles were the wrong publication type, for example, DNA methylation and PA were mentioned in the abstract, but the full-text screening revealed that it was a review paper rather than a primary study and did not include specific CpG site details. The final number of studies remaining for review was six (Figure 1; Table 2).

### 3.2. Participant Characteristics

All the participants were considered healthy with no known underlying health conditions, but there were variations in the baseline requirements of the populations selected for the studies. These included being classified as obese or a healthy weight, with BMI ranging from 26 to 32 kg/m^2^. PA levels were described as ranging from sedentary [48,51], light, moderate, moderate–vigorous and vigorous PA [47], trained [52], and low responders (LRE) and high responders (RES) to exercise [49]. There was also a wide age range between the studies, with age means spanning 23 to 63 years. Gender split varied, with female participants ranging from 0 to 63%. Participant characteristics for one cohort [50] were sourced from a previous study paper [53].

Baseline measurement methods varied between studies, with one reporting waist circumference [51] and two reporting waist-to-hip ratio [49,51]. The population study cohort was considerably larger than any of the PA intervention study cohorts. One study [49] used two distinct cohorts of LRE and RES, which have been treated separately for the purpose of this analysis. Table 3 provides a summary of the study population characteristics.

### 3.3. Study Design

Of the six studies selected, five were PA interventions and one was a population cohort validation study (Table 2). PA intervention durations varied between the shortest at four weeks [50] and the longest at six months [51], and load testing, which was not time-constrained [52]. Participant baseline measures taken prior to the PA intervention were used as an equivalent to a non-exercising control. Timings for sample collection varied between intervention studies, from three hours post-load testing [52] to one week after completing the final exercise session [50]. The types of exercise used in the PA interventions included resistance training [52] and endurance training [48,49,50,51]. One study compared two distinct cohorts for low responders and high responders to exercise [49], so both data sets were used for the purpose of this meta-analysis.

The population study [47] sought to validate results from a meta-analysis identifying CpG sites associated with PA using participant blood samples. This was completed using PA questionnaires and interviews to categorise participants into groups based on PA levels and calculated metabolic equivalents (METs), alongside a control population.

### 3.4. Study Quality Assessment Results

The studies were assessed for quality using the BIOCROSS method (Supplementary File Table S3). All scored between 15 and 17 points out of a possible 20, and all studies fulfilled at least one criterion of the three from each domain. As a higher score indicates a lower risk of bias, all studies could be considered as having a moderate-to-low risk of bias. All studies provided a clear rationale, objectives and detailed statistical analysis methods. However, none stated study power or rationale for sample size, one did not describe their participant inclusion or exclusion criteria [48] and only two mentioned their participation drop-off or completion rate [48,49].

### 3.5. Clinical Metrics

A range of clinical measures were used to establish the health of the cohorts pre- and post-intervention. Blood pressure was reported in three studies, although none reported significant improvements post-intervention [47,48,51]; another study [47] classified 46.7% of the cohort as hypertensive. Cholesterol levels and triglycerides were measured in three studies [47,48,51], with one study [51] recording a significant increase in high-density lipoprotein cholesterol (*p* = 0.02) post-PA. A range of glucose testing was conducted in four studies [47,48,49,51]; significant differences in insulin results were recorded in one study [49], including insulin fasting pmol/L (*p* = 0.013), insulin oral glucose tolerance test (*p* = 0.009), Matsuda insulin sensitivity indices (ISImats) (*p* = 0.001) and fasting serum insulin uIU/mL (*p* ≤ 0.001) in another study [48] post-PA. Glycated haemoglobin tests were conducted in three studies [48,49,51], and leukocytes and C-reactive protein were recorded in one [49], although no significant changes were identified post-PA.

Physical analyses included AT measures in one study [49], with a significant reduction in subcutaneous AT (*p* = 0.015), but not in visceral AT or total AT volume. One study [47] recorded PA levels using METs per week, and two measured exercise performance in watts (W) [48,49], with one study reporting significant differences in maximum W workload (*p* < 0.001) [48]. VO_2_ max was recorded in two studies [50,51], and lactate threshold and peak power in one [50]. VO_2_ peak was measured in one with significant differences post-intervention (*p* < 0.0001) [48]. Pre-intervention training metrics were reported for one cohort [52], including training age, squat tolerance, and muscle characteristics including vastus lateralis thickness and muscle fibre percentages; no measures were reported post-PA intervention.

**Figure 2:**
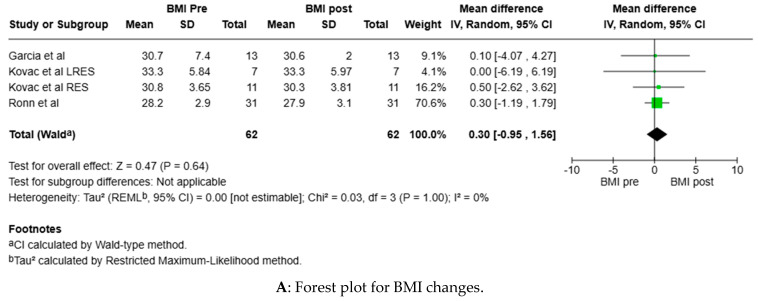

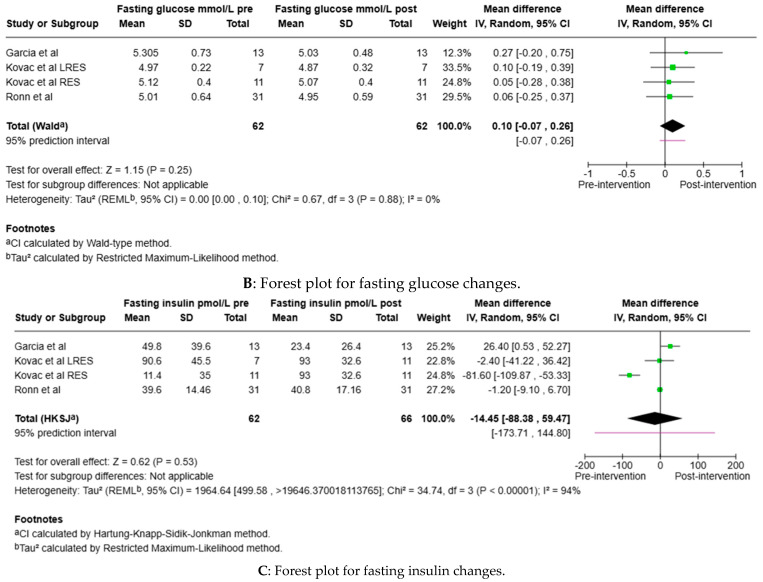
Forest plots for clinical measures taken before and after PA interventions. (**A**) BMI; (**B**) fasting glucose; (**C**) fasting insulin. *P* values indicate no significant changes in these clinical measures post-PA intervention. Statistical comparison was limited due to inconsistent reporting between studies.

Forest plots (Figure 2) were used to analyse changes in BMI, fasting glucose and fasting insulin before and after the PA intervention. Although individual studies reported some statistically significant changes as mentioned above, the overall effect changes were not found to have changed significantly post-intervention. Statistical comparisons between studies were limited, as very few studies reported on measures consistently.

### 3.6. Epigenomic Meta-Analysis

A range of tissue types were used for the DNAm analysis (Table 2). Four studies used SKM [48,49,50,52], one study used blood samples [47] and one study used AT [51]. Two SKM biopsies were taken at three and six hours following the short exercise bouts [52]; the data taken at six hours was used for this meta-analysis, as the other studies used longer timeframes for collection. All six studies used microarray analysis to identify CpG site methylation. Two studies used an Infinium HumanMethylation 450 bead chip [47,51] analysing 450 k CpG sites. Four studies used an Infinium MethylationEPIC 850 bead chip [48,49,50,52], analysing 850 k CpG sites, which included those sites covered by the Infinium HumanMethylation 450 bead chip [47].

DNAm was measured using either methylation β-values [49,51] or M-values [47,50]; one study reported M-values converted from β-values [52]. Four studies used *p*-values to identify significant results [47,48,49,52], and two used the false discovery rate (FDR), whilst also reporting *p*-values [50,51].

**Figure 3:**
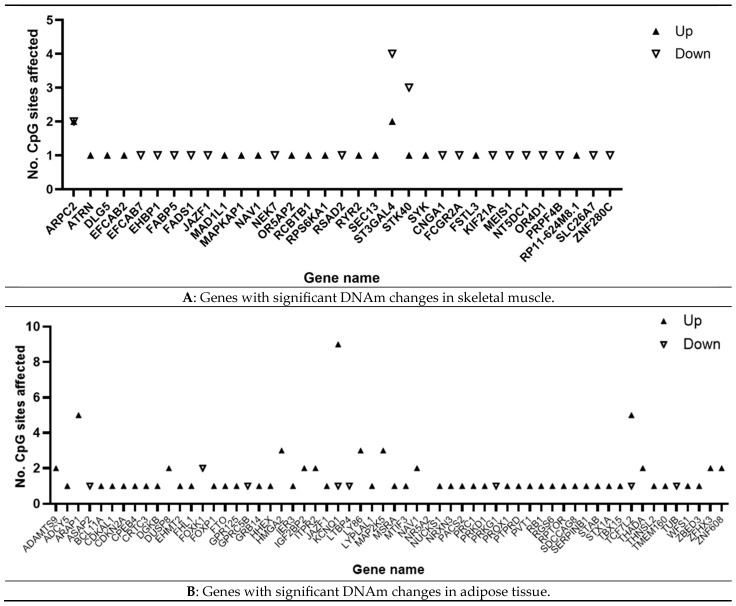
Genes with number of hyper/hypomethylated CpG sites associated with PA and obesity pathways post-PA. (**A**) Skeletal muscle; (**B**) adipose tissue. CpG sites located in genes associated with obesity-related pathways are shown. The direction of the triangle indicates whether DNAm had increased or decreased post-intervention.

A total of 257 CpGs reported as having DNAm significantly affected by PA across the six studies were compiled. Notably, 205 of these CpGs were located in 162 genes; of these CpGs, 134 were located in 92 genes associated with pathways related to obesity (Figure 3; Supplementary Information Tables S4–S6). CpG data were cross-checked between studies where available to understand if CpGs found significant in one study were analysed and found insignificant in other studies (Supplementary Information Table S7). Two studies agreed that genes JAZF1 and NAV1 [50,51] had CpG sites significantly affected by PA relating to obesity.

**Figure 4:**
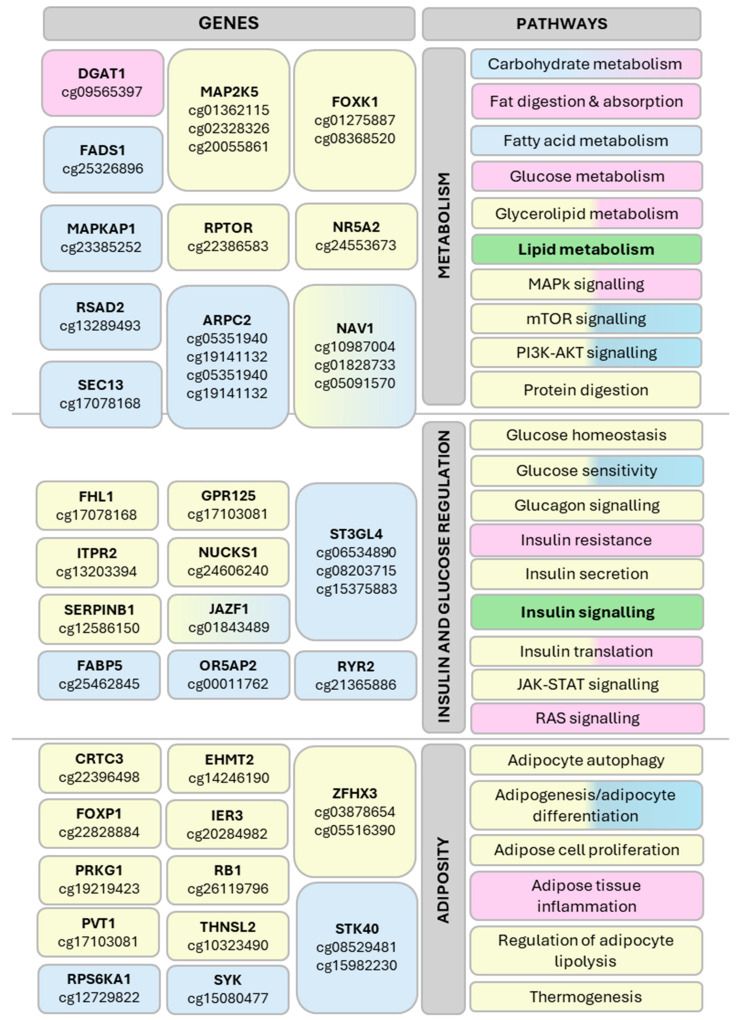
Pathways associated with CpG sites and genes related to obesity. Identified using EWAS [39] and KEGG [42] databases. Affected genes are grouped by association with obesity-related pathways involved with metabolism, insulin and glucose regulation and adiposity. Genes associated with more than one pathway grouping are shown overlapping the line. Colour code indicates tissue type as follows: Pink: blood; yellow: adipose tissue; blue: skeletal muscle; green: all three tissue types are represented.

Figure 4 provides a summary of significantly affected CpGs and genes with key pathway associations related to obesity, covering metabolism, insulin and glucose regulation and adiposity (Supplementary Information Table S8). Significantly affected genes were compared by tissue type, and JAZF1 and NAV1 were identified in both adipose tissue and skeletal muscle. Pathways were reviewed by tissue type, with insulin sensitivity and lipid metabolism identified as common pathways across all three tissue types.

## 4. Discussion

The current systematic literature review and meta-analysis was conducted to determine the effects of PA in healthy participants on DNAm profiles associated with obesity. This was completed by compiling results from DNAm studies of CpG sites in healthy adult populations, with varying levels of PA and body composition. The key findings of this research were that PA has an epigenetic influence on the DNAm of CpG sites and genes associated with obesity-related pathways. The genes that were differentially methylated in the current epigenomic meta-analysis belonged to the insulin sensitivity and lipid metabolism pathways and are involved with metabolic health.

DNAm and obesity studies have focused on weight loss resulting from a combination of lifestyle factor changes, including diet, alcohol consumption, smoking and PA [54], measuring success using phenotypic BC indicators such as BMI and waist circumference reduction. Although PA has been found to be more effective than diet alone [55], very few DNAm studies have focused on the effects solely of PA [19]. The studies identified in this meta-analysis were found to have a low risk of bias and could be considered of good quality.

Three of the selected studies noted a small reduction in BMI post-PA interventions [48,49,51], and one a significant increase in high density lipoprotein (HDL) [51], which indicate a change also observed in the molecular analysis. Excess adiposity is a hallmark of increased BMI, weight gain and obesity, and the epigenetic effect of PA on pathways associated with adiposity supports the principle of PA as an important factor in mitigating weight gain. In the current epigenomic meta-analysis, a total of 32 genes associated with adipogenesis and adipocyte differentiation were hyper methylated, including FOXP1, LYPLAL1 and TMEM160; FTO and MAP2K5 also have associations with energy and lipid metabolism [16]. One gene, TUB, was hypo-methylated, and two genes, STK40 and TCF7L2, were hypo-methylated on some CpG sites and hyper-methylated on others (Supplementary Table S8). This suggests that PA could help to mitigate weight gain through epigenetic changes in genes associated with pathways for lipid metabolism and adiposity.

Effective insulin action is an important mechanism in regulating blood glucose levels and energy storage, with insulin resistance being a risk factor for T2D. The glucose and insulin testing undertaken in two studies found significant clinical changes following their PA interventions [48,49], and there are multiple differentially methylated genes associated with insulin signalling identified in the molecular analysis. Two genes were identified in multiple studies, JAZF1 and NAV1 [50,51]. A total of 19 genes were hyper-methylated, including TCF7L2, and three genes were hypo-methylated (DGAT1, FABP5, GPRC5B); three genes, JAZF1, KCNQ1, and ST3GAL4, had both hyper- and hypo-methylation of CpG sites (Supplementary Table S8). These genes have an established link with the insulin signalling pathway and indirectly towards T2D disease pathology. Epigenetic changes in these genes indicate that PA can potentially mitigate T2D risk through insulin signalling pathways.

Raised blood pressure is an early indicator of CVD risk, and some measurements taken post-PA intervention indicate a reduction in systolic blood pressure [51] and diastolic blood pressure [48,51]. Differential methylation was observed in genes associated with vascular function and CVD, with three genes hyper-methylated (LY86, RBCTB1 and THNSL2), three genes hypo-methylated (FCCR2A, GPRC5B and PRKG1), and one gene, TCFL2, with both hyper- and hypo-methylated CpG sites (Supplementary Table S8). This suggests that PA can have a protective effect on CVD development through molecular pathways and physical health improvements.

Two genes of particular interest are NAV1 and JAZF1, which were identified in two different studies, and in both AT and SKM [50,51]. NAV1 is associated with the mammalian target of the rapamycin (mTOR) pathway, energy homeostasis control, and neurotransmitter signalling for gastrointestinal paths, including food intake and nutrient sensing [56]. Studies involving ablation of neurons expressing NAV1 in mice identified an exaggerated inflammatory response to a high-saturated fat diet, which suggests a role in limiting acute-phase response to dietary fat in an obesogenic diet [57]; responses differed by sex in mice on a high-fat, high-sugar diet, with males indicating weight gain resistance, while females were observed to have improved oral glucose tolerance and homeostasis, higher insulin levels and increased gastrointestinal transit [58]. JAZF1, juxtaposed with another zinc finger protein 1, plays a role in regulating several nuclear receptors and protein kinases involved in cellular energy metabolism processes [59], influencing downstream lipid and glucose homeostasis and inhibiting inflammatory response [60]. Overexpression of JAZF1 has been linked with reduced lipogenesis, increased lipolysis and a decrease in weight gain [61]. As obesity-associated chronic inflammation contributes to insulin resistance, JAZF1 has been identified as a potential target for therapeutic strategies for T2D due to its role in mitigating insulin resistance [60]. DNAm changes affecting CpG sites in genes involved with lipid metabolism and insulin sensitivity pathways were common in all three tissue types, indicating that PA does have an epigenetic effect on pathways associated with obesity.

The strengths of this systematic review and meta-analysis were the strict inclusion criteria for study selection, use of EWAS data and analysis of results by tissue type. Clinical metrics were aligned with genes and pathway findings, and genes of interest were identified for future research, including JAZF1 and NAV1. The laboratory methods for DNAm analysis were consistent across all six studies with the use of Infinium bead chip technology.

There were some limitations in the comparison due to the heterogeneity of the studies selected, with some confounding factors known to contribute to genetic and epigenetic variance, including diversity of cohorts for age [32] and sex [62] and limited information provided on ethnicity and economic background [27]. The design of PA interventions varied; resistance or endurance exercise type can affect DNAm profiles differently [63]. The timing of tissue sample collection post-PA should be considered, as there were variations in hours to days between the studies, which may have contributed to the heterogeneity of the results. Data from the load-testing biopsies indicated a higher level of DNAm changes after three hours than were identified at six hours compared to the pre-exercise baseline [52]; this could be attributed in part to an acute exercise-induced stress response [64], whereas long-term sustained exercise has been linked with the hypomethylation of genes associated with oxidative stress, such as OXR1, leading to a greater tolerance to oxidative stress in SKM [65]. This is reflected in the meta-analysis results, with the highest number of affected genes seen in the longest PA intervention [51], and the lowest number of affected genes in the short-bout load testing [52]. Epigenetic effects of repeated or sustained bouts of PA have been found to be more marked, suggesting that a cumulative effect of PA contributes to an epigenetic memory that may not be observed in a single PA intervention [52]. These are all factors that should be taken into consideration when designing a PA intervention study.

The results of this meta-analysis would benefit from further investigation and validation in a future cohort. The small number of studies that were selected and the heterogeneity of cohorts, PA intervention design and variations in DNAm value reporting and statistical analysis methods underline the need for more research in this area to build a more complete and consistent understanding of the epigenetic effects of PA on DNAm.

Understanding the epigenetic consequences of increased PA levels, and the impact this could have on metabolic health, highlights the importance of PA as a lifestyle intervention, independent of diet. The impact of PA on weight loss at a molecular level is an important contribution to our understanding of how lifestyle changes in this area can mitigate obesity-related health risks. Early intervention with PA initiatives can help to tackle weight gain and longer-term disease development such as T2D, CVD and other obesity-related health problems that have an impact on public health.

## 5. Conclusions

This study achieved the aims of identifying differentially methylated CpG sites and genes associated with obesity-related pathways as an epigenetic effect of PA. Multiple genes and pathways were identified as associated with BC and adiposity, which was supported by a reduction in BMI in some cohorts following PA intervention. Differential methylation was observed in genes associated with glucose and insulin pathways, with improvements also noted in clinical tests for insulin sensitivity, an important indicator for T2D. Finally, blood pressure changes in participants were observed, alongside DNAm changes seen in genes associated with blood pressure and CVD. This supports the idea that PA has important molecular consequences related to BC and obesity-related disease development such as T2D and CVD, independent of other lifestyle factors such as diet. Little research has been undertaken in this area so far, as evidenced by the small number and heterogenous nature of studies meeting our selection criteria, and our knowledge would benefit from more in-depth research to understand how PA can contribute to tackling the obesity challenge from an epigenetic perspective. Establishing a link between increased levels of PA and metabolic health improvements independent of diet underlines the importance of positive lifestyle changes that can contribute to weight loss and mitigate obesity-related health risks. PA as preventative medicine is a powerful message that could ultimately be used as part of a public health campaign, or to influence PA initiatives and funding at a local level.

## Figures and Tables

**Table 1:** PICOS framework used to establish eligibility criteria.

PICOS Element	Criteria
Population	Human population with no underlying health conditions, aged 18–65 years, non-smokers, pregnant or lactating excluded
Intervention	Assessment of DNA methylation resulting from: (1) PA levels; or (2) effects of PA programme
Comparison	(1) Control group for population study or (2) non-exercising group or participant baseline as control for PA programme
Outcome	DNA hypermethylation or hypomethylation in CpG sites
Study design	Population study or PA intervention study

**Table 2:** Summary of study characteristics.

Citation	Title	Country of Origin	Study Characteristics	Study Population	Study Numbers	Tissue Type	Publication	Risk of Bias Score (Table S3)
[47]	Physical activity and genome-wide DNA methylation: the REgistre GIroni del COR study.	Spain	Population study: validation of meta-analysis using PA questionnaires and blood sample analysis.	Existing cohort (REGICOR)	619; 5% female	Blood	American College of Sports Medicine	17
[48]	Can exercise training alter human skeletal muscle DNA methylation?	US	Exercise intervention: 8 weeks endurance training.	Sedentary healthy adults	13; 61% female	Skeletal muscle	Metabolites	15
[49]	Skeletal muscle gene expression signatures of obese high and low responders to endurance exercise training.	Germany	Exercise intervention: 8 weeks endurance training.	Healthy overweight adults	18; 63% female	Skeletal muscle	Journal of Clinical Endocrinology and Metabolism	17
[50]	Sex differences in muscle protein expression and DNA methylation in response to exercise training.	Australia	Exercise intervention: 4 weeks endurance training.	Healthy adults	78; 36% female	Skeletal muscle	BMC	16
[51]	A six-months exercise intervention influences the genome-wide DNA methylation pattern in human adipose tissue.	Sweden	Exercise intervention: 6 months endurance training.	Healthy middle-aged males	31; 0% female	Adipose tissue	PLOS Genetics	17
[52]	Skeletal muscle DNA methylation and mRNA responses to a bout of higher versus lower load resistance exercise in previously trained men.	US	Exercise intervention: resistance load testing, not time-constrained.	Active young males	11; 0% female	Skeletal muscle	Cells	16

**Table 3:** Study population characteristics.

Study/ Characteristic	[47]	[48]	RES [49]	LRE [49]	[50] *	[51]	[52]	Total/ Average
Number of participants	619	13	11	7	78	31	11	770 total
Mean age (yrs)	63.10 (11.70)	34.60 (11.10)	28.60 (4.72)	27.60 (3.96)	33.50 (7.50)	37.30 (4.40)	23.00 (4.00)	35.39 (6.76)
% female	49.90	61.00	54.50	71.40	35.90	0.00	0.00	38.96
Height (m)	NS	NS	1.72 (0.10)	1.71 (0.09)	NS	NS	1.80 (0.07)	1.74 (0.09)
Weight (kg)	NS	87.50 (24.10)	91.80 (17.10)	96.90 (17.30)	NS	91.80 (11.00)	86.00 (12.00)	90.80 (16.30)
BMI (kg/m^2^)	26.90 (4.00)	30.70 (7.40)	30.80 (3.65)	33.30 (5.84)	NS	28.20 (2.90)	27.00 (3.00)	29.4 (4.47)
Waist circumference (cm)	NS	NS	NS	NS	NS	97.70 (8.60)	NS	97.70 (8.60)
Waist-to-hip ratio	NS	NS	0.90 (0.05)	0.87 (0.05)	NS	0.93 (0.05)	NS	0.90 (0.05)

Compiled from the six studies considered in this review. Mean values for cohort populations at the start of the study. * Data sourced from previous Gene SMART cohort study [50,53]. (std dev) BMI = body mass index; NS = not stated.

## Data Availability

The original contributions presented in this study are included in the article/Supplementary Material. Further inquiries can be directed to the corresponding author(s).

## Supplementary Materials

The following supporting information can be downloaded at: https://www.mdpi.com/article/10.3390/ijerph22040637/s1, Table S1: Search strategy development process; Table S2: BIOCROSS domain scoring; Table S3: Outcome of BIOCROSS assessment; Table S4: CpGs, genes and pathway associations related to obesity; Table S5: Pathways with associated genes affected by CpG methylation identified in blood; Table S6: Pathways with associated genes affected by CpG methylation in skeletal muscle; Table S7: CpG sites cross-referenced across studies; Table S8: Genes with significant changes in DNAm associated with clinical measures [66,67,68,69,70,71,72,73,74,75,76,77,78,79,80,81,82,83,84,85,86,87,88,89,90,91,92,93,94,95,96,97,98,99,100,101,102,103,104,105,106,107,108,109,110,111,112,113,114,115,116,117,118,119,120,121,122,123].

## Abbreviations

The following abbreviations are used in this manuscript:

AT	Adipose tissue
BC	Body composition
BMI	Body mass index
CpG	Cytosine–phosphate–guanine
CVD	Cardiovascular disease
DNA	Deoxyribonucleic acid
DNAm	DNA methylation
EWAS	Epigenome-wide association studies
FDR	False discovery rate
GWAS	Genome-wide association studies
LRES	Low responders to exercise
MET	Metabolic equivalents
NICE	National Institute for Health and Care Excellence
PA	Physical activity
RES	High responders to exercise
RNA	Ribonucleic acid
SB	Sedentary behaviour
SKM	Skeletal muscle
T2D	Type II diabetes
WHO	World Health Organisation
