# Supplementary material for: Molecular Effects of Physical Activity and Body Composition: A Systematic Review and Meta-Analysis
Source: Int J Environ Res Public Health. 2025 Apr 18;22(4):637. doi: 10.3390/ijerph22040637 (PMC12026539; doi:10.3390/ijerph22040637)
Supplement: Supplementary file 1 [file ijerph-22-00637-s001.zip › ijerph-3504743-supplementary.pdf]

Supplementary materials

Table S1: Search strategy development process.

Search no.	Search terms	Database					
		**Pubmed/ Medline**	**SPORT Discus**	**Embase**	**Scopus**	**Cochrane Library**	**Web of Science**
(17)	(“epigenetic” OR “genetic” OR “biomarker” OR “methylation” OR “DNAm” OR “CpG”) AND (“physical activity” OR “physical exercise” OR “PA”) AND (“body composition” OR “BMI” OR “body mass index” OR “waist circumference”)	2,235	121	5,298	5,632	2	3,743
(16)	“epigenetic” OR “genetic” OR “biomarker” OR “methylation” OR “DNAm” OR “CpG”	1,143,338	6,050	848,677	2,823,193	467	1,530,013
(15)	“CpG”	25,036	98	15,429	43,509	3	39,025
(14)	“DNAm”	1,229	2	1,188	1,485	53	1,552
(13)	“methylation”	76,389	206	43,116	156,329	38	143,018
(12)	“biomarker”	147,487	812	163,934	490,878	118	210,993
(11)	"genetic"	960,049	4,981	682,063	2,286,444	323	1,168,178
(10)	“epigenetic”	62,687	208	24,966	75,278	1	78,457
(9)	“physical activity” OR “physical exercise” OR “PA”	390,256	38,344	501,159	412,761	582	1,608,408
(8)	“PA”	275,112	8,925	367,679	208,220	374	1,433,009
(7)	“physical exercise”	11,693	1,843	9,816	19,992	46	18,004
(6)	“physical activity”	122,137	32,292	142,164	207,820	187	185,878
(5)	(“body composition” OR “BMI” OR “body mass index” OR “waist circumference”)	325,175	17,171	434,193	422,244	140	364,365
(4)	“waist circumference”	29,209	1,360	51,404	54,424	16	32,944
(3)	“body mass index”	243,506	10,976	238,965	280,674	117	234,417
(2)	“BMI”	151,695	6,004	267,732	196,852	89	156,410
(1)	“body composition”	45,535	6,042	44,944	77,970	22	66,481

Table S2: BIOCROSS domain scoring. Each section contained three questions requiring a yes/no response if the components had been included in the study [37].

Domain	Domain section	Maximum points
1. Study rationale	a. Hypothesis/objective	2
2. Design/methods	a. Study population selection	2
	b. Study population representativeness	2
3. Data analysis	a. Study population characteristics	2
	b. Statistical analyses	2
4. Data interpretation	a. Interpretation and evaluation of results	2
	b. Study limitations	2
5. Biomarker measurement	a. Specimen characteristics and assay methods	2
	b. Laboratory measurement	2
	c. Biomarker data modelling	2
**Total possible points**		**20**

Table S3: Outcome of BIOCROSS assessment. Each study was rated against the five domains and section criteria (supplementary Table S2) with a possible maximum total score of 20.

	Domain/section score										
**Study**	**1a**	**2a**	**2b**	**3a**	**3b**	**4a**	**4b**	**5a**	**5b**	**5c**	**Total**
Fernandes-Sanles et al, 2020 [47]	2	2	1	1	2	2	2	2	1	2	**17**
Garcia et al, 2022 [48]	2	1	1	1	1	2	2	2	2	1	**15**
Kovac et al, 2023 [49]	2	2	1	1	2	2	2	2	1	2	**17**
Landen et al, 2023 [50]	2	1	1	1	2	2	2	2	1	2	**16**
Ronn et al, 2013 [51]	2	2	1	1	2	2	2	2	1	1	**17**
Sexton et al, 2023 [52]	2	2	1	1	2	2	2	2	1	1	**16**

Table S4: CpGs, genes and pathway associations related to obesity. Indicating the gene and CpG sites affected. Up indicates hypermethylation, down indicates hypomethylation and the number of CpG sites affected where there are methylation differences within the same gene. Tissue type: AT = adipose tissue; SKM = skeletal muscle.

Gene	CpG	Up/ down	Pathway associations	Tissue type	Study identified
ADAMTS9	cg05501868; cg21527616	Up	Insulin sensitivity	AT	[51]
ADCY5	cg14567877	Up	BMI and obesity	AT	[51]
ARAP1	cg03720898; cg06838038; cg10495997; cg15279866; cg27058763	Up	Insulin signalling	AT	[51]
ARPC2	cg05351940; cg19141132; cg05351940; cg19141132	2 up, 2 down	Insulin sensitivity, protein digestion and absorption, oxidative phosphorylation, thermogenesis, calcium signalling	SKM	[47]
ASAP2	cg21821308	Down	Vitamin D receptor pathway	AT	[51]
ATRN	cg02693745	Up	Melanocortin signaling pathways regulating energy homeostasis; inflammatory response	SKM	[48]
BCL11A	cg01865786	Up	Insulin signalling	AT	[51]
CDKAL1	cg03390300	Up	Insulin signalling	AT	[51]
CDKN2A	cg07562918	Up	Insulin signalling	AT	[51]

CNGA1	cg03825843	Down	Phototransduction; cGMP-PKG signalling pathway; cAMP signalling pathway	SKM	[43]
CPEB4	cg07233933	Up	Adiposity	AT	[51]
CRTC3	cg22396498	Up	Adipogenesis	AT	[51]
DGAT1	cg09565397	Down	Triglyceride biosynthesis, insulin resistance, glycerolipid metabolism, fat digestion and absorption	Blood	[45]
DGKB	cg20836993	Up	Adiposity	AT	[51]
DLG5	cg01849559	Up	Hippo signalling	SKM	[48]
DUSP8	cg01602287; cg26902557	Up	Insulin signalling	AT	[51]
EHMT2	cg14246190	Up	Adipogenesis, inflammatory response, lypolysis	AT	[51]
FABP5	cg25462845	Down	Insulin and glucose sensitivity	SKM	[48]
FADS1	cg25326896	Down	Fatty acid metabolism	SKM	[48]
FCGR2A	cg27565811	Down	Atherosclerosis and CVD	SKM	[43]
FHL1	cg05455393	Up	Insulin sensitivity	AT	[51]
FOXK1	cg01275887; cg08368520	Down	Regulation of hepatic lipid metabolism	AT	[51]
FOXP1	cg22828884	Up	Adipocyte differentiation and thermogenesis, glucose homeostasis	AT	[51]
FTO	cg26580413	Up	Adiposity	AT	[51]
GPR125	cg17103081	Up	Glucose homeostasis, secretion of insulin	AT	[51]
GPRC5B	cg09141413	Down	Insulin signalling	AT	[51]
GRB14	cg22380033	Up	Insulin signalling	AT	[51]
HHEX	cg20180364	Up	Insulin signalling	AT	[51]
HMGA2	cg16965605; cg17182048; cg17518348	Up	Adiposity	AT	[51]
IER3	cg20284982	Up	Adipogenesis	AT	[51]
IGF2BP2	cg06150454; cg13918631	Up	Insulin signalling	AT	[51]
ITPR2	cg13203394; cg07645296	Up	Insulin secretion, glucagon signalling	AT	[51]
JAZF1	cg01843489; cg02963803	1 up, 1 down	Lipid and carbohydrate metabolism	SKM, AT	[50,51]
KCNQ1	cg01689159; cg03660952; cg04894537; cg06838584; cg08160246; cg13577072; cg15910264; cg19672982; cg24725201; cg25786675	8 up, 2 down	Insulin signalling	AT	[51]
LY86	cg02212836; cg05021589; cg09249494	Up	Insulin signalling	AT	[51]
LYPLAL1	cg16681597	Up	Adiposity	AT	[51]
MAD1L1	cg21543346	Up	Cell cycle control	SKM	[50]
MAP2K5	cg01362115; cg02328326; cg20055861	Up	Adiposity	AT	[51]
MAPKAP1	cg23385252	Up	mTOR signalling	SKM	[50]

MSRA	cg27519910	Up	Adiposity	AT	[51]
MTIF3	cg20147645	Up	Adiposity	AT	[51]
NAV1	cg10987004 (SKM); cg01828733 (AT); cg05091570 (AT)	Up	mTOR signalling	SKM; AT	[50,51]
NEK7	cg11521274	Down	pro-inflammatory interleukins (IL)-1beta and IL-18	SKM	[50]
NR5A2	cg24553673	Up	mapK signalling pathway	AT	[51]
NRXN3	cg16420308	Up	Adiposity	AT	[51]
NUCKS1	cg24606240	Up	Insulin signalling	AT	[51]
OR4D1	cg12463722	Down	Olfactory transduction	SKM	[48]
OR5AP2	cg00011762	Up	Insulin sensitivity	SKM	[50]
PACS2	cg00808648	Up	Mitochondria associated membranes	AT	[51]
PRC1	cg04775232	Up	Adiposity	AT	[51]
PRKD1	cg16592301	Up	Adiposity	AT	[51]
PRKG1	cg19219423	Down	Regulation of lipolysis in adipocytes, vascular muscle function	AT	[51]
PROX1	cg01902845	Up	Adiposity	AT	[51]
PTPRD	cg14545834	Up	Adiposity	AT	[51]
PVT1	cg00555695	Up	Adipogenesis, fatty acid synthesis/oxidation	AT	[51]
RB1	cg26119796	Up	Adipocyte differentiation (adipogenesis)	AT	[51]
RCBTB1	cg11801959	Up	DNA damage/repair, CVD contributor	SKM	[50]
RGS6	cg01775802	Up	G-protein signalling (modulates opioid response to fatty/sugary food intake)	AT	[51]
RPS6KA1	cg12729822	Up	Regulation of adipogenesis and adipocyte autophagy	SKM	[50]
RPTOR	cg22386583	Up	MTOR signalling	AT	[51]
RSAD2	cg13289493	Down	Lipid metabolism, cholesterol biosynthesis	SKM	[50]
RYR2	cg21365886	Up	Calcium release channels	SKM	[50]
SDCCAG8	cg16104450	Up	Adiposity	AT	[51]
SEC13	cg17078168	Up	Nucleocytoplasmic transport; Protein processing in endoplasmic reticulum; mTOR signalling pathway; Amyotrophic lateral sclerosis	SKM	[50]
SERPINB1	cg12586150	Up	Immune response; lipid metabolism, insulin sensitivity	AT	[51]
SLC26A7	cg06442162	Down	Gastric acid secretion	SKM	[48]
ST3GAL4	cg06534890; cg08203715; cg15375883	2 up, 4 down	Insulin sensitivity	SKM	[47]
STAB1	cg08222913	Up	Adiposity	AT	[44]
STK40	cg08529481; cg15982230	1 up, 3 down	Adipogenesis	SKM	[49]
STX1A	cg09871057	Up	Glucose tolerance	AT	[51]
SYK	cg15080477	Up	Adipocyte differentiation (adipogenesis)	SKM	[50]
TBX15	cg26104752	Up	Adiposity	AT	[51]
TCF7L2	cg00831931; cg05923857; cg06403317; cg09022607; cg19226647; cg23951816	5 up, 1 down	Adiposity	AT	[51]
THADA	cg01649611; cg12277798	Up	Adiposity	AT	[51]
THNSL2	cg10323490	Up	Adiposity (women only)	AT	[51]

TMEM160	cg19694781	Up	Adiposity	AT	[51]
TUB	cg05003666	Down	Adiposity	AT	[51]
WFS1	cg16417416	Up	Insulin signalling	AT	[51]
ZBED3	cg22051204	Up	Insulin signalling	AT	[51]
ZFHX3	cg03878654; cg05516390	Up	Adiposity (adipogenesis)	AT	[51]
ZNF280C	cg25445870	Down	Transcription factor for muscle tissue; link to T2D and CVD	SKM	[48]
ZNF608	cg01610165; cg12817840	Up	Adiposity	AT	[51]

Table S5: Pathways with associated genes affected by CpG methylation. Identified in blood [47].

**Pathway**	**Genes**
Adrenergic signaling in cardiomyocytes	RYR2
Autophagy - other	RPTOR
Axon guidance	PARD6G; ROBO2
B cell receptor signaling pathway	SYK
Bacterial invasion of epithelial cells	ARPC2; CTTN
Base excision repair	MBD4
Biosynthesis of unsaturated fatty acids	FADS1
cAMP signaling pathway	CNGB3
Cell cycle	RB1
Cellular senescence	ITPR2; RB1
cGMP-PKG signaling pathway	CNGA1
Dilated cardiomyopathy	RYR2
Dopaminergic synapse	ITPR2
Endocrine resistance	RB1
Endocytosis	ARPC2; ASAP2
Metabolic pathways	DGAT1; EHMT2; FADS1; HAL; PLD3; ST3GAL4
MicroRNAs in cancer	FOXP1; RPTOR
Motor proteins	KIFC1
Retrograde endocannabinoid signaling	SLC17A6
Ribosome	RPS7
Ribosome biogenesis in eukaryotes	NVL
Serotonergic synapse	ITPR2
Shigellosis	ARPC2; CTTN; ITPR2; RPTOR
Signaling pathways regulating pluripotency of stem cells	ZFHX3

Table S6: Pathways with associated genes affected by CpG methylation. Identified in skeletal muscle studies [48–50,52].

**Pathway(s)**	**Genes**
Alzheimer Disease	ADAM10; ITPR2; SNCA
Amphetamine addiction	STX1A
AMPK signaling pathway	RPTOR
Amyotrophic lateral sclerosis	SEC13
Apelin signaling pathway	ITPR2; RYR2
Apoptosis	ITPR2
Arrhythmogenic right ventricular cardiomyopathy	RYR2
Bladder cancer	RB1
Breast cancer	RB1

Calcium signaling pathway	ITPR2; RYR2; PTK2B
cAMP signaling pathway	CNGA1; RYR2; BDNF
Cardiac muscle contraction	RYR2
Cell adhesion molecules	CLDN5
cGMP-PKG signaling pathway	ITPR2; PRKG1
Complement and coagulation cascades	CLU
Coronavirus disease - COVID-19	RPS7; SYK
Cortisol synthesis and secretion	ITPR2
Cytoskeleton in muscle cells	FHL1
Diabetic cardiomyopathy	RYR2
Human papillomavirus infection	PARD6G
Human T-cell leukemia virus 1 infection	CRTC3; MAD1L1; RB1; SPI1
Huntington Disease	BDNF; STX1A
Hypertrophic cardiomyopathy	RYR2
Inflammatory mediator regulation of TRP channels	ITPR2
Influenza A	RSAD2
Insulin resistance	RPS6KA1
Insulin secretion	STX1A; TRPM4
Insulin signaling pathway	RPTOR
JAK-STAT signaling pathway	FHL1
Kaposi sarcoma-associated herpesvirus infection	ITPR2; RB1; SYK
Leukocyte transendothelial migration	CLDN5
Osteoclast differentiation	SPI1; SYK
Oxytocin signaling pathway	ITPR2; RYR2
Pancreatic cancer	RB1
Pancreatic secretion	ITPR2; RYR2
Parathyroid hormone synthesis, secretion and action	ITPR2
Parkinson disease	ITPR2; SNCA
Pathogenic Escherichia coli infection	ARPC2; CLDN5; CTTN
Phototransduction	CNGA1
PI3K-Akt signaling pathway	RPTOR; SYK; BDNF
Platelet activation	ITPR2; PRKG1; SYK
Polycomb repressive complex	FOXK1
PPAR signaling pathway	FABP5
Prion disease	ITPR2; RYR2
Progesterone-mediated oocyte maturation	MAD1L1; RPS6KA1
Prostate cancer	RB1
Protein processing in endoplasmic reticulum	SEC13
Proteoglycans in cancer	CTTN; ITPR2
Rap1 signaling pathway	PARD6G
Ras signalling	BDNF
Regulation of actin cytoskeleton	ARPC2
Regulation of lipolysis in adipocytes	PRKG1
Renin secretion	ITPR2
Renin-angiotensin system	ACE
Retinol metabolism	DGAT1
Retrograde endocannabinoid signaling	ITPR2
Small cell lung cancer	RB1
SNARE interactions in vesicular transport	STX1A
Thermogenesis	RPTOR; SMARCA4
Thyroid hormone synthesis	ITPR2
Tight junction	ARHGEF18; ARPC2; CLDN5; CTTN; PARD6G
Transcriptional misregulation in cancer	SPI1
Tuberculosis	SYK

Vascular smooth muscle contraction	ITPR2; PRKG1
Viral carcinogenesis	MAD1L1; RB1; SYK
Virion - Hepatitis viruses	CLDN5
Yersinia infection	ARPC2

Table S7: CpG sites cross-referenced across studies. The six studies specifically stating CpGs identified were cross-referenced. The CpG sites identified as having methylation significantly affected by PA in one study were checked against CpG data provided in the other studies to establish whether they were analysed, and not reported as significantly affected by PA.

Study	No. CpG sites identified as significant	No. CpG sites analysed by other studies and found not significant				
		[47]	[48]	[49]	[50]	[51]
[47]	2	-	2	2	2	0
[48]	11	0	-	9	3	0
[49]	7	0	7	-	6	0
[50]	92	0	91	92	-	0
[51]	119	0	109	112	104	-

Table S8: Genes with significant changes in DNAm associated with clinical measures. Genes highlighted in bold indicate that there were various CpG sites that were hyper methylated or hypo methylated within the same gene.

Area affected	Genes hyper methylated	Genes hypo methylated
Adipogenesis and adipocyte differentiation	ADCY5 [51,66], CPEB4 [51,67], CRTC3 [51,68], DGKB [51,69], EHMT2 [51,70], FOXP1 [51,71], FTO [51,72], HMGA2 [51,73], IER3 [51,74], LYPLAL1 [51,75], MAP2K5 [51,76], MSRA [51,77], MTIF3 [51,78], NRXN3 [51,79], PRC1 [51,80], PRKD1 [51,81], PROX1 [51,82], PTPRD [51,83], PVT1 [51,84], RB1 [51,85], RPS6KA1 [50,86], SDCCAG8 [51,87], STAB1 [51,88], **STK40** [49,89], SYK [50,90], TBX15 [3,51], **TCF7L2** [51,91] THADA [51,92], THNSL2 [51,93], TMEM160 [51,94], ZFHX3 [51,95], ZNF608 [51,96]	**STK40** [49,89], **TCF7L2** [51,91], TUB [51,97]
Insulin action	ADAMTS9 [51,98], ARAP1 [51,99], BCL11A [51,100], CDKAL1 [51,92], CDKN2A [51,92], DUSP8 [51,101], FHL1 [51,102], GPR125 [51,103], GRB14 [51,104], HHEX [51,92], IGF2BP2 [51,99], ITPR2 [51,105], **JAZF1** [51,59], **KCNQ1** [51,106], LY86 [51,107], NAV1 [51,108], NUCKS1 [51,109], SERPINB1 [51,110], **ST3GAL4** [49], STX1A [51,111], WFS1 [51,112] and ZBED3 [51,113]	DGAT1 [47,114], FABP5 [50,115], GPRC5B [51,116], **JAZF1** [51,59], **KCNQ1** [51,106], **ST3GAL4** [49]
Blood pressure	LY86 [51,117], RCBTB1 [50,118], **TCF7L2** [51,119], THNSL2 [51,120]	FCGR2A [48,121], GPRC5B [51,122], PRKG1 [51,123], **TCF7L2** [51,119]
